# Supplementary material for: Identification of YWHAH as a Novel Brain-Derived Extracellular Vesicle Marker Post Long-Term Midazolam Exposure during Early Development
Source: Cells. 2023 Mar 22;12(6):966. doi: 10.3390/cells12060966 (PMC10047367; doi:10.3390/cells12060966)
Supplement: Supplementary file 1 [file cells-12-00966-s001.zip › !Supplemental files/BDEV Supplemental Figures.pdf]

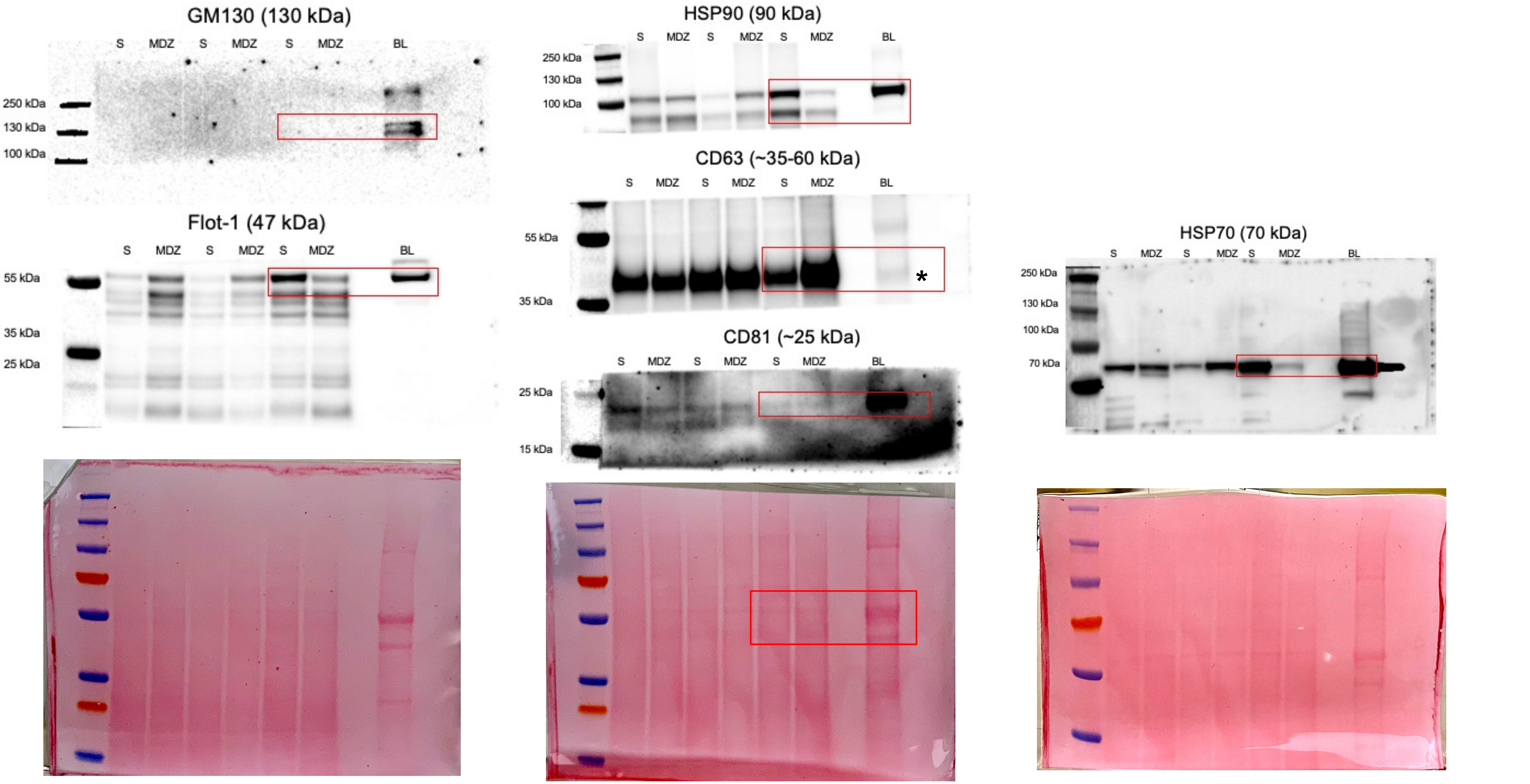

Supplement Figure S1. Validation of BDEV markers. Individual western blots on isolated BDEVs were used in the study. Ponceau-stained membranes are at the bottom for each membrane. Boxed blots are shown in Figure 1 of the manuscript. S-Saline, MDZ-Midazolam, BL-Brain Lysate

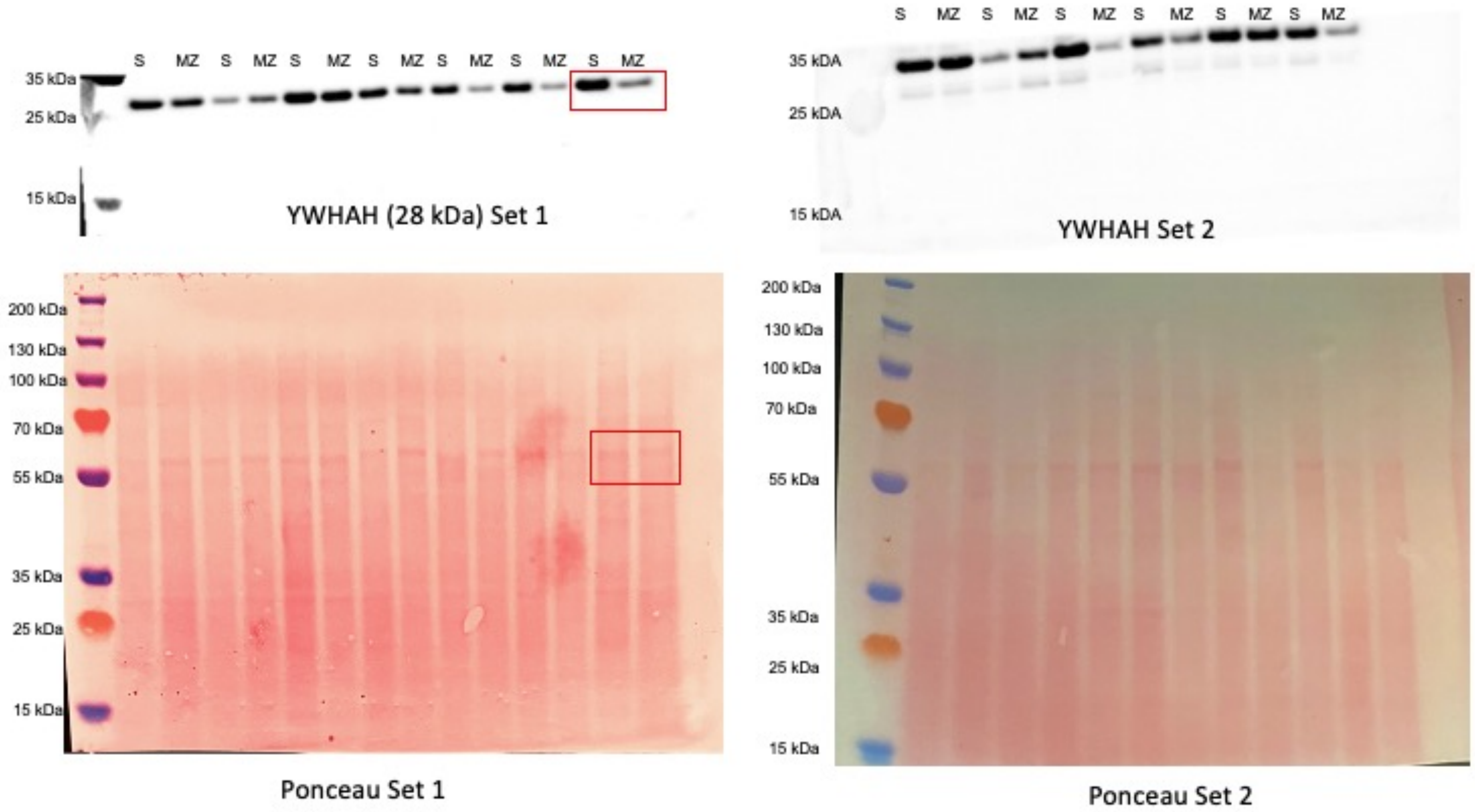

Supplement Figure S2. Individual western blots on isolated BDEVs in all 13 BDEV samples/groups used in the study. Boxed blots are shown in Figure 7 of the manuscript. S-Saline, MZ-Midazolam.
